# What is the relationship between viral prospecting in animals and medical countermeasure development?

**DOI:** 10.1128/mbio.02033-25

**Published:** 2025-08-25

**Authors:** Aishani V. Aatresh, Marc Lipsitch

**Affiliations:** 1 Harvard College 124049 , Cambridge, Massachusetts, USA; 2 Center for Communicable Disease Dynamics, Harvard T.H. Chan School of Public Health 552346 , Boston, Massachusetts, USA; 3 Department of Epidemiology, Harvard T.H. Chan School of Public Health 189346 , Boston, Massachusetts, USA; 4 Department of Immunology and Infectious Diseases, Harvard T.H. Chan School of Public Health 1857 , Boston, Massachusetts, USA; Tsinghua University, Beijing, China

**Keywords:** preparedness, surveillance, zoonoses, spillover, medical countermeasures, vaccines, virus families, Ebola, Marburg

## Abstract

In recent decades, surveillance in non-human animals has aimed to detect novel viruses before they “spill over” to humans. However, the extent to which these viral prospecting efforts have enhanced outbreak preparedness remains poorly characterized, especially in terms of whether they are necessary, sufficient, or feasible to spur vaccine development. We find that several viruses which pose known threats to people lack approved vaccines, and known viruses discovered in human patients before 2000 have caused most major 21st-century outbreaks. With *Filoviridae* as a case study, we show there is little evidence that viral prospecting has accelerated vaccine or drug development or that systematically discovering novel zoonotic viruses in animal hosts before they cause human outbreaks has been feasible. These results suggest that surveillance for novel viral zoonoses does not accelerate vaccine development and underscore questions about its importance for outbreak preparedness. We consider limitations to these conclusions and alternative approaches to preparedness and response.

**IMPORTANCE:**

Sampling in animal populations to detect novel viruses before they infect humans has been a major activity justified by several considerations, notably by the idea that finding such viruses will stimulate the development of medical countermeasures such as vaccines. This article examines the evidence that such research leads to earlier vaccine development and finds the evidence lacking. This is important because, in an era of scarce resources and biosafety considerations for researchers, efforts should be directed to those activities most likely to yield the desired outcomes.

## INTRODUCTION

In recent decades, concerns about viruses with the potential to “spill over” from non-human animals to humans and cause major outbreaks have driven efforts to characterize viral diversity and discover novel viral species in animal hosts (1–4). The COVID-19 pandemic intensified discussions about isolating from animals currently unknown zoonotic viruses, before these viruses might spill over to humans and cause a large outbreak of “Disease X” (5–8). To this end, surveillance in animals, especially wildlife, has been central to One Health proposals in renewed efforts toward pandemic prevention, preparedness, response, and resilience (9–11). Yet some elements of viral surveillance projects in animals remain poorly defined, namely: what assumptions motivate them, how knowledge of viral zoonoses in animals has structured preparedness and response, and whether assumptions and expected benefits have proven realistic.

Approximately 250 viruses are known to infect humans; zoonotic spillover is the proximate cause for an estimated 60% of emerging infectious disease events, of which about 70% originate in wildlife (12). Examples of emerging infectious disease events—defined in this estimate as the first occurrence of a pathogen in a human population, an increase in geographic range or incidence for previously known pathogens, or major changes in pathology—range from Crimean-Congo hemorrhagic fever (CCHF) and Nipah virus infection to yellow fever and Ebola virus disease. The remaining approximately 40% of events were caused by other types of pathogens (e.g., bacterial or fungal infections that did not involve a non-human host, including drug-resistant existing bacteria), pathogens of unknown origin, or increased human-to-human transmission of a known virus.

The potential to understand the extent of unknown viral diversity and dynamics of viral ecology and disease emergence has motivated viral discovery in animal hosts. One study posits that over 600,000 unknown viruses with the potential to infect humans lurk in birds and non-human mammals (13); another more recent estimate suggests that 10,000 viruses with zoonotic potential circulate in non-human mammals (14). Proponents aim to use this information before outbreaks in humans to accelerate and focus follow-on interventions, build technical capacity, and reduce human-wildlife contact to improve global preparedness for such outbreaks. PREDICT is one notable example of a viral discovery program that systematically sampled animals for viruses of future relevance. It was led by the United States Agency for International Development and the University of California, Davis from 2009 through 2020, with partnerships in over 30 countries. This project focused on “[p]redicting where new diseases may emerge from wild animals and detecting other pathogens before they spread among people [to] give us the best chance to prevent new pandemics” by advancing an “approach in which pathogens of pandemic potential are discovered at their source before large-scale epidemics occur in people” (15). PREDICT detected over 800 novel viruses in samples (from approximately 7,000 animals) tested across 15 taxonomic families with known viruses that infect humans; some novel viruses were strains of known viruses based on pairwise sequence identity cut-offs (16). However, project meta-analyses note that total effort—more than 500,000 samples primarily from over 70,000 animals—yielded just one novel viral species known to be capable of causing disease in humans, and it was discovered while research teams were supporting the investigation of an outbreak in humans (17, 18).

We term these systematic predictive efforts for “finding viruses in wildlife before they emerge in humans” *viral prospecting* to distinguish discovery and forecasts of future disease threats from capacity-building related to surveillance for known viruses, another major aim of PREDICT-style programs. In the discovery and forecasting vein, some researchers have argued that viral prospecting benefits not only basic research and international development but also efforts to predict disease emergence and accelerate medical countermeasure (MCM) development for those diseases (3, 13, 19–23). One author “envisage[s] countries working together to fund viral discovery programs that upload sequence data in almost real time, so that it can be used to identify those microbes most likely to be able to cause zoonoses, and the data then can be used to block spillover and create vaccines” (24). Having vaccines in particular at-the-ready during an outbreak has become increasingly central in 21st-century infectious disease preparedness discussions, with the idea that development prior to possible large-scale human outbreaks will reduce morbidity, mortality, and economic consequences (25–27).

To date, assessments of viral prospecting efforts have focused primarily on the distribution of and disease burden in host and reservoir species (4, 28), sampling and viral discovery curves (29–31), cost-benefit analyses (32), and predicting the likelihood of spillover for different viruses (16, 33, 34). Critiques have revolved around safety risks and, to a lesser extent, the significance of discovered viruses for human health. Biosecurity or biosafety has been invoked to justify the termination of already funded large-scale prospecting projects (35–38); literature documents sampling efforts during which wildlife biologists were bitten by animals known to host viral zoonoses, sometimes causing infection (39, 40). Other commentaries focus on mitigating zoonotic spillover (10, 41) or highlight concerns about the feasibility of prospecting efforts and consequences of unmet expectations for trust in science (42). This literature pays comparatively less attention to the potential translational impacts of viral discovery. Many factors beyond the isolation of a novel pathogen influence MCM development; the effect of one intervention (i.e., surveillance to detect novel viruses in animals) cannot be isolated, and sampling constraints limit evaluations of these efforts. Despite these limitations and the termination of some existing projects in recent years, critical reflection can inform deliberations about preparedness strategies that involve viral prospecting and MCM development.

This analysis specifically considers the claim that viral prospecting promotes the development of MCMs, particularly vaccines—which are widely discussed as one of the most tangible instruments and outputs for 21st-century preparedness efforts and directly intended to prevent and mitigate disease mortality and spread. We assess the extent to which viral prospecting, especially in wildlife, has enabled vaccine development to strengthen preparedness and response for outbreaks in humans. Where other studies weigh costs and benefits, we focus on understanding these possible benefits: what would need to be true for them to be realized as suggested, and to what extent have foundational assumptions and projections materialized over time?

We address these questions by testing two primary hypotheses: (i) prospecting in animals identifies potential (pre-emptive) and actual (post-hoc) viral causes of outbreaks in humans, and (ii) it translates to accelerated vaccine development. We identify and assess related conditions that would need to hold true to justify arguments for the necessity, sufficiency, and feasibility of viral prospecting in animals vis-à-vis vaccine development: (i) past efforts at viral prospecting have paid off in that scientists preemptively discovered in zoonotic hosts viruses which later caused disease in humans; (ii) the discovery of a virus in an animal host prior to the first outbreak in humans has translated to enhanced preparedness and response, defined primarily through available vaccines; (iii) novel viruses are causing outbreaks and plausibly could have been discovered in an animal before these outbreaks; (iv) viruses prioritized for vaccine development were discovered in animal hosts; and (v) viral prospecting supplies needed candidates for vaccine development because efforts to address most known viral threats are underway and succeeding. We evaluate these hypotheses and assumptions through case studies across virus families, with an in-depth exploration of *Filoviridae*, and consider limitations of these analyses alongside possible alternative approaches.

## RESULTS

### The discovery of viruses prior to the first documented outbreaks in humans has had a limited effect on preparedness and response

We first evaluated which viruses known to cause human disease were discovered in animals and whether the initial discovery in an animal host or vector has substantially improved preparedness and response. We identified 11 viruses that were isolated in animals prior to causing clusters of cases in humans, out of approximately 250 viruses known to infect humans (Table 1). Three viruses—monkeypox, Rift Valley Fever (RVF), and Zika—have caused notable outbreaks in humans since their discovery. Knowledge of these viruses from an animal source prior to their first outbreaks in humans has not translated to distinctively robust or sufficient capacity to prevent or respond to future outbreaks; each has spread beyond previously contained regions (43–45). Furthermore, no vaccine is licensed for use in humans against Zika or Rift Valley Fever, and those approved for monkeypox virus are, at the time of writing, repurposed or expanded-use smallpox vaccines (46). The other viruses on this list cause seemingly limited disease in humans as detected through often-scant diagnostic and surveillance capacity.

**TABLE 1:** Viruses first discovered in animals before causing an outbreak in humans

Virus	Discovered	First documented human outbreak	Conditions of isolation and description
Barmah forest	1989	1992	*Culex* mosquitoes in Australia
Bunyamwera/Cache Valley	1943	1955	Through yellow fever surveillance in Uganda; subtype isolated in Utah mosquitoes (1956)
Eastern equine encephalitis	1831	1938	North American horses
Monkeypox	1958	1970	Research monkeys; first observed human infection in a child in the Democratic Republic of the Congo. Zoonotic reservoir unknown.
Ngari	1979	1993	*Aedes simpsoni* mosquitoes in Senegal
Puumala	1979	2000	Bank voles in Finland
RVF	1931	1975	Outbreak on a sheep farm in Kenya
Semliki forest	1942	1987	*Aedes abnormalis* mosquitoes in western Uganda
Sindbis	1952	1961	*Culex* mosquitoes in the Nile River Delta, Egypt; used as a model alphavirus in USA
Venezuelan equine encephalitis	1938	1950	From the brain of a deceased horse
Zika	1947	1952	Through yellow fever surveillance in infected rhesus macaques for research use

### Twenty-first-century outbreaks of international concern largely have been caused by known viruses first discovered in humans

**Fig 1:**
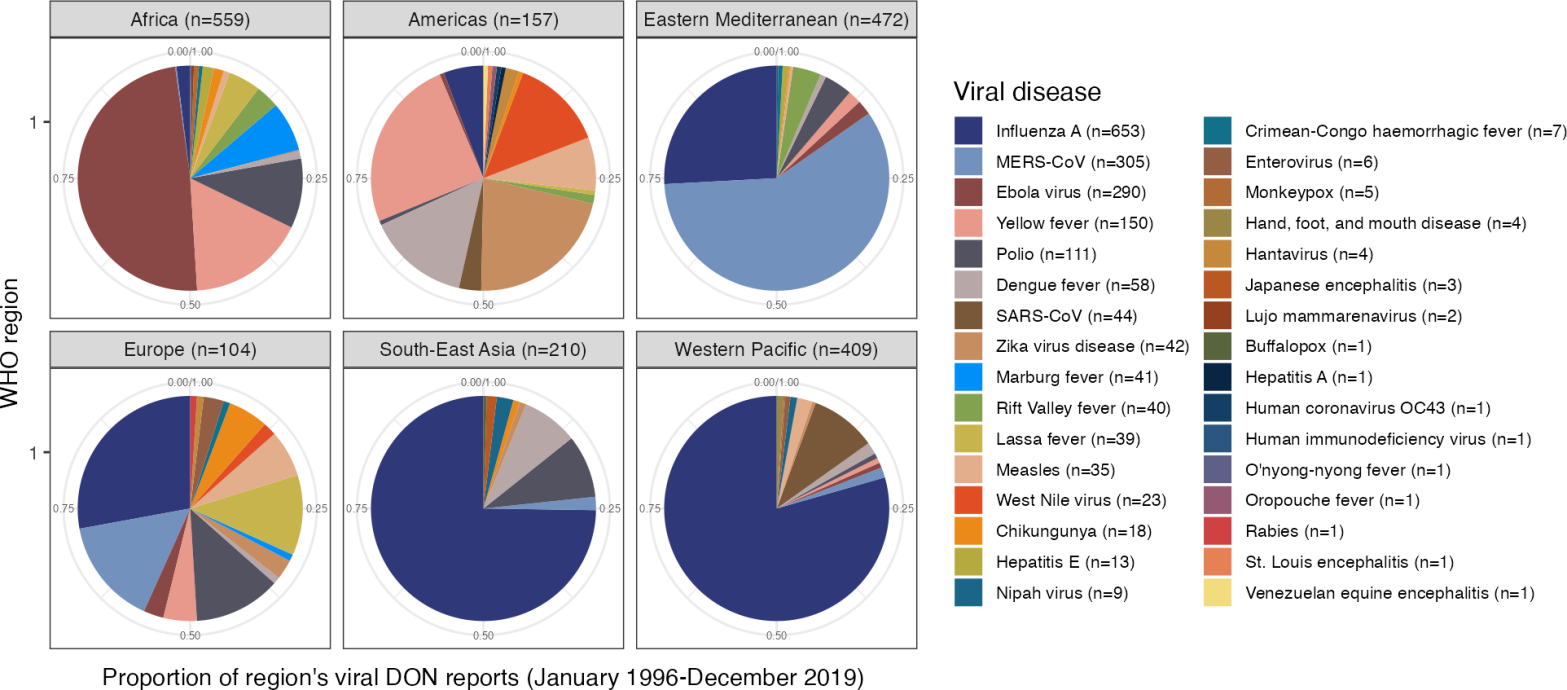
Geographic and viral distribution of WHO DON reports (1996–2019). Regional distribution for virus-only subset of WHO DON reports from Carlson et al. (47) database. Major contributors—Africa: Ebola virus, yellow fever, and polio; Americas: dengue fever, Zika virus disease, yellow fever, and West Nile virus; East Mediterranean: MERS-CoV and influenza A; Europe: influenza A, Lassa fever, and measles, MERS-CoV, and polio; South-East Asia: influenza A, dengue fever, and polio; Western Pacific: influenza A and SARS-CoV.

We next assessed whether high-consequence outbreaks in humans have been caused by then-novel viruses that could have been discovered in animals. Data from the World Health Organization’s (WHO) Disease Outbreak News (DON) system and Public Health Emergency of International Concern (PHEIC) declarations suggest otherwise. Since 1996, DON has been WHO’s public online system to compile and disseminate disease event reports from countries and partner organizations. Using Carlson et al.’s 1996–2019 DON database as a proxy for general trends in viral disease events (47, 48), we found that a small number of known viruses account for the majority of internationally reported disease events (Fig. 1). Across regions, the plurality of DONs involves Ebola virus, influenza A, MERS-CoV, and yellow fever. These distributions largely reflect extended and widespread outbreaks of known viruses or spikes in endemic disease. Fewer DON reports document spillovers of novel viruses (e.g., initial emergence of MERS-CoV and Nipah virus).

DON reports on-the-ground early warning signals of potentially emerging outbreaks. In contrast, PHEIC declarations reflect a formal committee evaluation that a disease event has grown to pose a widespread international threat (49). WHO has declared seven PHEICs since establishing the instrument in 2005 (Table 2). PHEICs primarily have been associated with viruses that were well characterized or had caused outbreaks in humans prior to the emergency in question. In the two events (H1N1 and COVID-19) of a novel strain or species leading to a PHEIC, the pathogen belonged to an extensively studied virus family. These data suggest that spillovers of novel zoonotic pathogens are not driving outbreaks that countries report to WHO or that international public health experts deem most emergent, rendering viral prospecting for novel threats at best insufficient to advance preparedness for international disease concerns.

**TABLE 2:** WHO PHEIC declarations by viral disease and discovery status

Year	Viral disease
2009	Influenza A/H1N1 (triple reassortant)^*a*^
2014	Ebola
2014	Polio (*ongoing PHEIC*)
2016	Zika
2018	Ebola
2020	COVID-19^*a*^
2022	Mpox

^*a*^ Novel pathogen.

### Viral prospecting makes limited contributions to MCM development pipelines

The analyses described in the preceding sections characterize the viruses causing disease in humans and their relationship to viruses discovered in animal hosts. Next, we next assess whether viral discovery in animal hosts addresses a rate-limiting step in vaccine development efforts. We aimed to determine if viral prospecting has discovered pathogens of interest for global efforts to develop vaccines against epidemic threats and if scientists lack pathogens worth targeting to these ends, such that viral prospecting might be necessary to expand vaccine development horizons.

**Fig 2:**
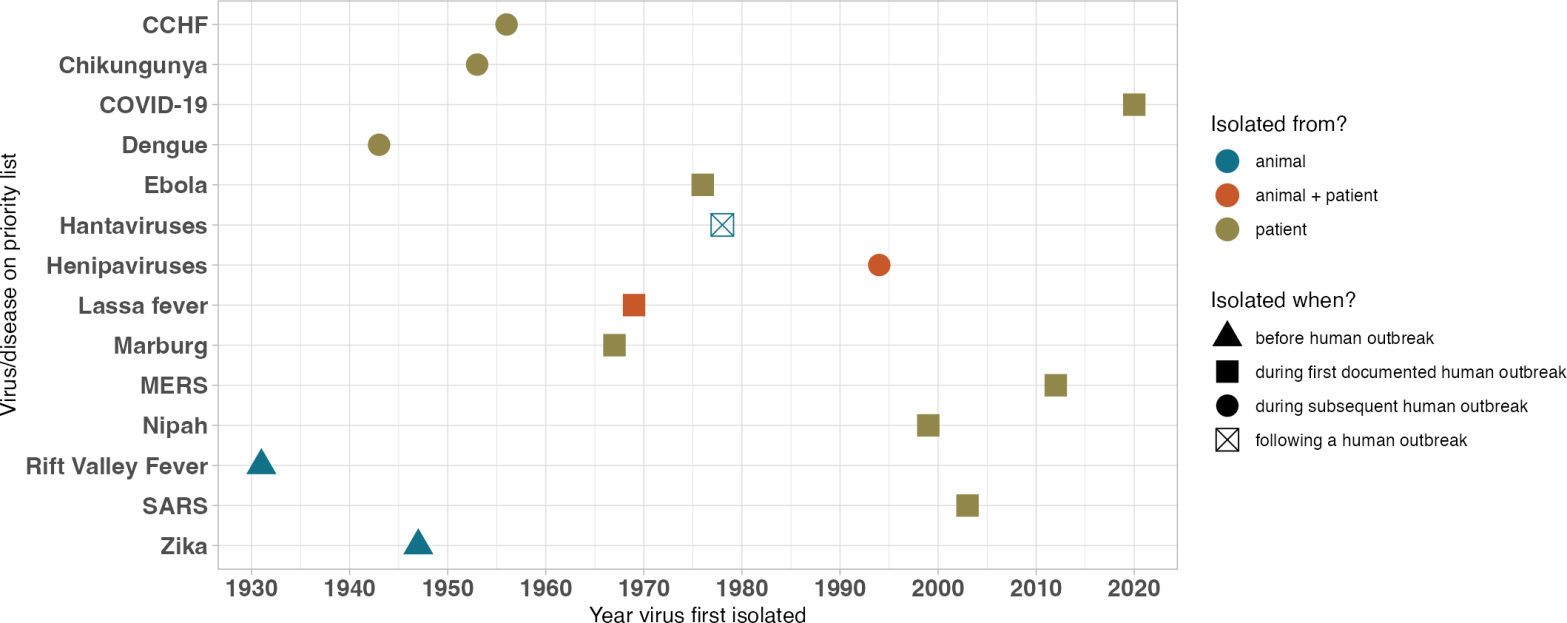
Characteristics of viruses on priority lists for MCM research and development. Priority pathogens for research under WHO, NIAID (category A [as of December 2023]), UKVN, and/or CEPI were assessed for mode of transmission, first recorded outbreak, and circumstances under which they were first isolated. “First documented human outbreak” is differentiated from “during a human outbreak” to note pathogens that were isolated after the first known instance of disease in humans.

Since 2018, four major institutions have released or updated comprehensive priority lists for development of MCMs, especially vaccines: the Coalition for Epidemic Preparedness Innovations (CEPI), U.S. National Institute of Allergy and Infectious Diseases (NIAID), U.K. Vaccine Network (UKVN), and WHO (50–53). We find that viruses prioritized for MCM development by at least one agency predominantly were first isolated well before 2000 and during a virus’ first documented outbreak in humans (Fig. 2). Systematic 21st-century viral prospecting efforts have not contributed any novel zoonotic viruses to these lists; viral discovery in animals more broadly has played a limited role, largely via viruses isolated from mosquitoes.

We also characterized the vaccine development landscape for known viral threats. By some classifications, 26 virus families contain at least one virus known to infect humans (54). For the majority of these families, the first virus isolated was from humans between 1940 and 1980 (Table 3). At present, a regulatory body has approved at least one vaccine for use in humans against at least one virus in 16 families (14 before 2020). The first vaccine approved for a coronavirus was against SARS-CoV-2 during the COVID-19 pandemic. This vaccine relied on years of research and development efforts following outbreaks of SARS-CoV and MERS-CoV in humans. The first vaccine for a pneumovirus was approved in 2023, for respiratory syncytial virus (RSV). Of the 10 known families for which currently no vaccine is approved for any virus, some absences are striking. The lack of a human vaccine for any retrovirus is notable as HIV is a prominent retrovirus that has afflicted humans for decades. Also striking is the absence of a bunyavirus vaccine; Rift Valley Fever, Crimean-Congo hemorrhagic fever, and hantaviruses are long-established disease threats from the family. In summary, significant gaps remain in vaccine development against known targets. Some, like HIV, have been the focus of widespread efforts and have posed significant technical challenges. Zoonotic viruses discovered in animal hosts have contributed few previously unknown targets of interest to further direct these efforts.

**TABLE 3:** Vaccine development and viral discovery across virus families^*a*^

Family	Year first vaccine approved for human use	Year first virus in family isolated, source	Notes	Examples of notable viruses
Adenoviridae	1971	1953, human	Only vaccine against an adenovirus approved for use in the US military; separate from adenoviruses used as vectors for vaccines against other pathogens	
Anelloviridae	–	1997, human	Estimated >90% prevalence in population, primarily nonpathogenic	
Arenaviridae	2006	1933, human	2006 Junin virus vaccine, only licensed for use in Argentina	Lassa fever
Astroviridae	–	1975, human		
Bornaviridae	–	1975, animal	Horses and sheep extensively vaccinated	
Bunyavirales	–	1931, animal	Veterinary vaccine used extensively in Africa for RVF	RVF, CCHF^*b*^, and Hantavirus
Caliciviridae	–	1972, human	Cats vaccinated; no human concerns beyond norovirus	
Coronaviridae	2020	1965, human		SARS-CoV-2, SARS-CoV, and MERS-CoV
Filoviridae	2019	1967, human		Ebola and Marburg
Flaviviridae	1937	1927, human		Dengue, Zika, Japanese encephalitis, and yellow fever
Hepadnaviridae	1981	1965, human		Hep B
Hepeviridae	2011	1990, human	Vaccine licensed in China and Pakistan	Hep E
Herpesviridae	–	1919, human		
Orthomyxoviridae	1945	1933, human		Influenza
Papillomaviridae	2006	1979, human		HPV
Paramyxoviridae	1963	1934, human		Measles, mumps, and Nipah
Parvoviridae	–	1960, animal	Some disease in humans but usually asymptomatic; human trials stopped for severe adverse events. Family established 1975. Human disease first observed 1974.	
Picobirnaviridae	–	1988, human	Opportunistic enteric pathogens	
Picornaviridae	1955	1898, animal		Polio
Pneumoviridae	2023	1956, animal	Separated from *Paramyxoviridae*, 2016	RSV
Polyomaviridae	–	1953, animal		
Poxviridae	1796	–	Edward Jenner and cowpox-based vaccination for smallpox	Smallpox and monkeypox
Reoviridae	1998	1953, human		Rotavirus
Retroviridae	–	1908, animal		HIV
Rhabdoviridae	1885	–	Louis Pasteur and inactivated rabies virus vaccine	Rabies
Togaviridae	1969	1930, animal	Separated from *Flaviviridae*, 1984	Rubella

^*a*^ “–” indicates no vaccine approved for human use.

^*b*^ CCHF-Crimean-Congo hemorrhagic fever.

### Viral discovery in animals plays a limited role in MCM development and outbreak response for filoviruses that infect humans

Two genera of *Filoviridae*, *Orthoebolavirus* and *Orthomarburgvirus*, include zoonotic viral species that have caused several outbreaks in humans over the past half-century. The 2013–2016 Ebola epidemic in West Africa is the largest filovirus outbreak to date, with several clinical trials for MCMs amidst over 28,000 cases and 11,000 deaths (55). Since the first international reports of filovirus disease in humans from the 1970s, extensive resources have been devoted to detecting filoviruses in animals. In 2022 and 2023, outbreaks of Ebola virus disease (EVD) and Marburg virus disease (MVD) occurred in sub-Saharan African countries where cases had not been previously documented. Therefore, filoviruses offer a case study in which growing concern about outbreaks in humans has been complemented by relatively widespread sampling efforts in animals and MCM research and development.

**Fig 3:**
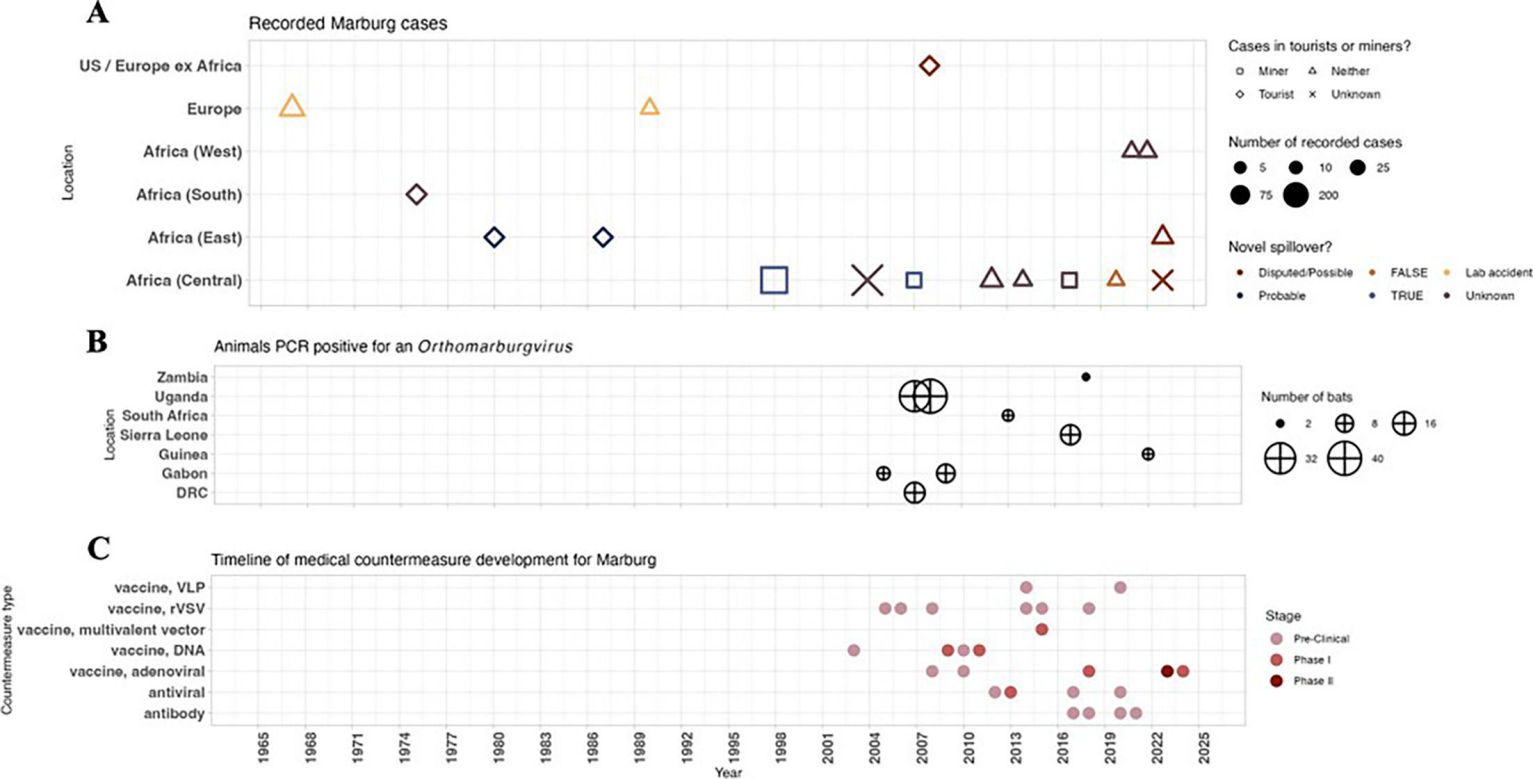
Marburg outbreaks, animal surveillance, and MCM development. (**A**) Timeline of MVD outbreaks by country, assessment of novel spillover event as source of outbreak, and primary outbreak population. The Angola outbreak, the largest recorded, is the large “X” at 2004 and Africa (Central). (**B**) Timeline of when and where samples from animals were positive for Marburg virus by PCR. (**C**) Timeline of published pre-clinical (and non-murine) work and clinical trials by type of MCM specifically targeting Marburg virus.

The first filovirus cases in humans were documented in 1967 during two simultaneous Marburg virus outbreaks in Germany and then-Yugoslavia; both were traced back to infected laboratory African green monkeys (Fig. 3A). The next several Marburg cases were reported predominantly in tourists who visited caves that were known bat habitats in African national parks. Other outbreak investigations have found either tenuous or no epidemiological or genomic linkages to bats as a putative source of infection (Supplementary Information). The largest MVD outbreak to date began in Angola in 2004, infecting over 250 people and causing more than 225 deaths. Before that was a 1998 outbreak in the Democratic Republic of the Congo (DRC); it revealed years of internationally unreported possible MVD with over 50 cases documented in local records of “a disease called ‘syndrome hémorragique de Durba,’ which was always associated with mining, [and] was common knowledge among villagers and health care workers in the area” (56).

In 2007, animal surveillance definitively established Egyptian fruit bats as a Marburg reservoir by isolating live virus from several animals (Fig. 3B). This discovery was preceded by 16 samples from bats in caves in the DRC and Gabon and followed by 40 samples obtained in Uganda from August 2008 through December 2009 that were PCR positive for an *Orthomarburgvirus*. These discoveries have been followed by a limited number of samples positive for an *Orthomarburgvirus* that causes disease in humans.

Despite decades of outbreaks in humans and extensive animal surveillance efforts, no approved vaccine or therapeutic specifically targets MVD, and only one has progressed past phase I trials (Fig. 3C). Concerted pre-clinical and clinical-stage MCM development followed the Angola outbreak, with a plurality of DNA and viral vector vaccine candidates designed using isolates from infections during this outbreak (Supplementary Information). Research for vaccine and drug candidates has progressed during and after subsequent human outbreaks, without any distinctive relationship to viral discovery in animal hosts, even upon the discovery of a novel *Orthomarburgvirus* strain through PREDICT (57).

**Fig 4:**
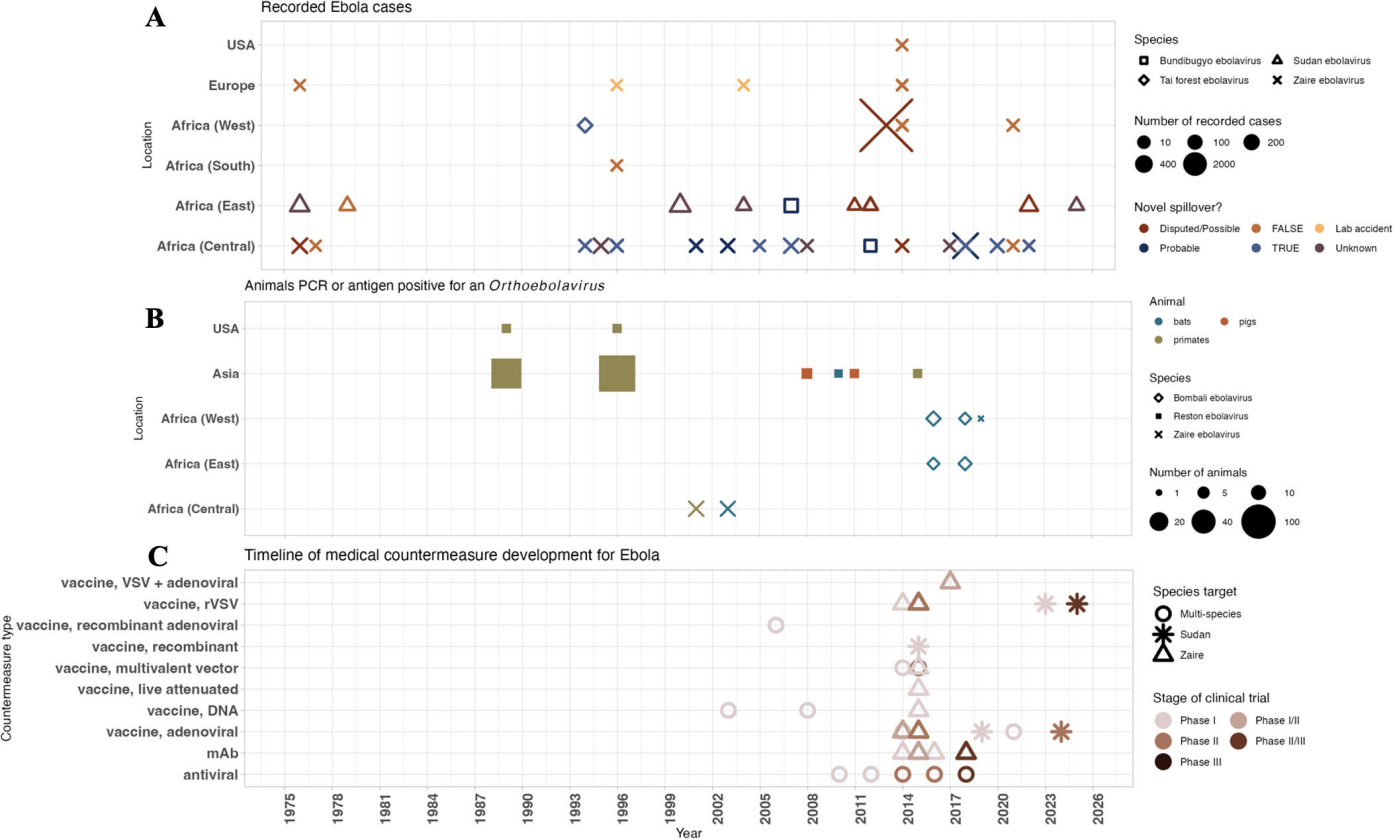
Ebola outbreaks, animal surveillance, and MCM. (**A**) Timeline of EVD outbreaks by species, assessment of novel spillover event as source of outbreak, and location. (**B**) Timeline of when and where samples from animals were positive for an *Orthoebolavirus* by PCR or antigen test. (**C**) Timeline of clinical trials by type of MCM specifically targeting an *Orthoebolavirus*.

EVD outbreaks, animal surveillance, and MCM development present similar patterns. Scale-up of MCM development primarily followed the historic 2013–2016 West Africa epidemic; animal surveillance has found limited success detecting any *Orthoebolavirus* that causes disease in humans (Fig. 4). The Zaire species has caused most EVD outbreaks since the first documented cases in 1976 (Fig. 4A). Several outbreak origins investigations have offered inconclusive evidence for the source of disease or implicated flare-ups of undetected human-to-human transmission (Supplementary Information). Some studies of the 2013–2016 epidemic undermine the hypothesis that the epidemic originated with a spillover event from a bat to a child near a tree in the Guinean village of Meliandou, where nonetheless samples from bats have not yielded *Orthoebolavirus* genetic material (58). Interviews with villagers suggest that rather than a bat, a persistently infected survivor from Sierra Leone in close contact with the child and his family may have transmitted the virus (59).

Simultaneously, viral discovery in animals has done little to prospectively identify novel viruses that cause EVD in humans. In 2016, PREDICT discovered an *Orthoebolavirus* species, which was named the Bombali virus (Fig. 4B) and appears able to enter human cells (60). However, *in vitro* and *in vivo* studies suggest Bombali has low pathogenic potential in humans; it has not caused documented infections in humans (61, 62). Furthermore, no Bombali-specific MCM development has progressed to a pre-clinical phase, and no MCM has specific regulatory approval for use against Sudan ebolavirus (Fig. 4C). MCM development for *Orthoebolavirus* until recently focused on the Zaire species, though Sudan ebolavirus vaccines have been evaluated in clinical trials during recent resurgences.

## DISCUSSION

Our analysis finds that viral prospecting in animals has played a limited role in advancing vaccine-related preparedness and response for viral diseases in humans. Our findings raise questions about whether viral prospecting is necessary, sufficient, or feasible to predict emerging infectious diseases and drive MCM development related to them.

If prospecting measures were necessary to prompt MCM development for important targets, we would expect to find that few high-value candidates for such development are already known. On the contrary, numerous viruses and viral families that cause disease in humans lack vaccines, and most disease outbreaks in humans involve known human pathogens or close relatives of them. No vaccines are approved for use in humans against any virus in 10 of 26 virus families known to infect humans. Within the 16 of 26 virus families that contain at least one virus with an approved vaccine, several known threats (e.g., Zika, West Nile, Nipah, and Lassa) do not have a vaccine approved for widespread use. This suggests that knowledge of a virus is insufficient to advance vaccine research and development to a stage that markedly enhances the likelihood that a vaccine will be available at the inception of a disease emergency. Instead, most priority viruses for current MCM development efforts are pathogens that were discovered several decades ago but remain “high-value” targets with a lack of effective interventions, for reasons ranging from technical challenges to market failures (63), and larger regional outbreaks have been the catalyzing force for accelerated vaccine R&D. Licensure, rather than a first-in-human trial, is the endpoint for this analysis because phase 1 trials often do not raise the financial and technical challenges that complicate scaled-up studies and production of vaccines; time to regulatory authorization in an emergency is an increasingly major focus for international epidemic preparedness and response efforts (64–66).These data do not account for early-stage research for vaccine candidates, often funded by various national funding agencies. However, they illustrate that R&D efforts have not exhausted the work required to translate from known viral threats to approved vaccines against them.

If viral prospecting were sufficient to prompt MCM development for important targets, we would expect that, historically, pathogens discovered in animals before human cases would have prompted MCM development. Instead, we find that for the 11 viruses to our knowledge discovered in a zoonotic host before documented clusters of cases in humans, there have been few differences or advances in capacity for preparedness and response relative to viruses first discovered during a human outbreak. Most of these viruses have not caused major outbreaks in humans, but three viruses have caused several significant outbreaks; MCM development against them has not progressed differently from viral threats discovered in other ways. While mosquito-based surveillance has identified several viruses with the ability to cause disease in humans and enabled various ecological and virological studies during and prior to outbreaks, systematic surveillance in wildlife or domestic animals—the focus of most viral prospecting efforts today—has been comparatively less successful.

If viral prospecting were a feasible way to spur MCM development against important targets, we would similarly expect to see historical examples where identification of animal reservoirs for viruses that could or do infect humans has prompted MCM development. Instead, searches for viruses in animals have been generally inadequate for pre-emptive and sometimes even post-hoc identification of viral threats to humans. Prospecting efforts in animals to date have had limited success in identifying novel viruses likely to cause substantial outbreaks in humans. Animal surveillance has achieved limited success in isolating from wildlife, especially bats, PCR- or antigen-positive samples of viruses that cause disease in humans. It has isolated only one novel Ebola or Marburg species (*Orthoebolavirus bombaliense*), which seems as-yet unable to cause outbreaks in humans—as with other novel filoviruses discovered through viral prospecting (67, 68)—and has received scant attention as a target for MCM development (69, 70). Intensive efforts to definitively establish animal reservoirs for known *Orthoebolaviruses* have fallen short. Our analyses of filoviruses also highlight a larger body of research regarding viruses with known zoonotic hosts or assumed reservoirs that sometimes reemerge due to undetected or changing human-to-human transmission rather than new spillovers (71–78). Given the difficulties associated with retrospectively discerning the origins of known outbreaks, the case is tenuous for prospectively identifying the source of a possible future outbreak from an animal reservoir to advance preparedness. Surveillance in animals to find viruses with a high risk for spillover presumes that we have valid and reliable predictors of spillover probability (79). Our inability to identify the source of known outbreaks suggests that we do not have the data required for such predictors. Viral prospecting does not address disease emergence from repeated spillovers of known viruses and flare-ups of previously undetected human transmission, although repeat spillovers from known reservoirs point to the potential utility of some veterinary vaccines (80).

These analyses have limitations, including an exclusive focus on viral pathogens. First, we explore filoviruses as a case study to offer a concrete and tractable analysis of zoonotic viral discovery and MCM development for pathogens that so far have had only regional transmission but are not significantly “neglected.” Lessons from this case may not generalize across virus families. Filoviruses present a scenario where MCM development has been framed through biosecurity and concerns historically smaller but plausibly under-detected outbreaks. They do not pose the same challenges of respiratory transmission, mutation and reassortment rates, or panzoonotic disease dynamics that more strongly may motivate viral discovery efforts for coronaviruses or orthomyxoviruses (influenza). Second, our analysis mainly addresses regulatory approval or lack thereof for the use of a vaccine in human populations, but it neither evaluates capabilities for scaled-up, widespread MCM manufacturing and equitable access nor accounts for veterinary vaccine development. Third, we make no explicit cost-benefit or risk-reward assessments. Finally, this analysis focuses on viral prospecting to identify novel threats rather than zoonotic surveillance as a broader category of efforts to characterize viral diversity, build technical capacity, or inform behavioral interventions and basic research. It, relatedly, does not account for less tangible ways in which prior knowledge about viruses (81), general healthcare system strengthening, or non-pharmaceutical interventions have factored into outbreak responses. We do not equate the lack of direct MCM development with a lack of value for virus prospecting.

One response to the study findings is that they underscore the need for more extensive viral prospecting and MCM development efforts by highlighting the shortcomings of limited resources and efforts thus far. However, there are demonstrated difficulties with robustly assessing the pathogenicity of all viruses discovered through such initiatives and various bottlenecks in translating knowledge of a virus to a usable vaccine or therapeutic. Scaling viral discovery would not address these translational gaps. The fact that existing discoveries have not prompted significant MCM efforts reinforces the view that viral prospecting is not the rate-limiting step. A second response is that increased animal surveillance to discover new viruses would aid efforts to manage and curb human-wildlife interactions (e.g., bushmeat hunting) and stop possible spillover events before they even occur, especially in developing countries (82). However, the realities of an unequal and globalized world complicate these proposals. For example, bats are common in various habitats and can be a source of subsistence for some communities in the global South (83), while several internationally driven development projects have disrupted ecosystem dynamics in ways that have exacerbated disease risk and changed how people interact with animals (84). On the other hand, intensive agricultural practices or various factory farming-related risks of spillover from animal hosts in countries like the United States have gone somewhat unattended (85, 86), such that responses to recent zoonotic outbreaks, such as H5N1 from cattle, have suffered from a lack of coordinated infrastructure (87). To mitigate the risks of spillover, it is essential to interrogate and address the social and political dimensions of the contexts in which diseases emerge (88).

Therefore, one possible implication of our analysis is that preparedness for emerging viruses might focus less on populating an “atlas” (89) of potential viral zoonotic threats and more on attending to under-considered existing phenomena (e.g., endemic viruses, public health infrastructure, and socioeconomic disparities). Mpox, the disease associated with the monkeypox virus, offers an additional illustrative example. Decades of increased disease incidence in parts of Africa (90, 91) attracted little international attention until a widespread global epidemic and PHEIC in 2022. One recent study suggests that what was once thought to be spillover-induced spread of disease was, in fact, undetected human-to-human transmission (92). The first country in Africa received doses of the smallpox-monkeypox vaccine in August 2024, more than 2 years after many other parts of the world (93). The discovery of monkeypox in an animal prior to the first documented outbreak of the disease in humans has had limited bearing on these contemporary challenges. As others have proposed, more focused serological and viral surveillance for disease in humans who closely interact with wildlife or livestock might provide more effective proxies for emerging disease risks and a clearer picture of disease burden while furthering knowledge of viral ecology (42, 94). Furthermore, organizations like CEPI were created to address vulnerabilities posed by market failure-related gaps in R&D. These approaches may contribute more concretely and reliably to preparedness against future known and unknown viral threats, whether through vaccine development projects or other related initiatives that mobilize dedicated technical, financial, and political resources. Current trends in American politics to reduce or eliminate such commitments notably compromise these efforts.

In summary, viral prospecting in non-human animals does little to detect novel disease threats of consequence for societies across the world and has little to show in terms of advancing translational research for vaccines. Notwithstanding the value of acquiring further knowledge about viral diversity and expanding scientific capacity, narrow demonstrated benefits raise questions about whether other modes of preparedness might offer more suitable ways of achieving similar and further ends, without additional tradeoffs in cost, safety, or other domains. Closer attention to people’s lives and known viral diseases—whether emergent, endemic, or neglected—might produce strategies that more comprehensively address inequalities, baseline capacity, and governance related to routine health needs and cross-boundary phenomena, simultaneously strengthening preparedness and response against future emerging outbreaks.

## MATERIALS AND METHODS

Analyses of viruses that cause disease in humans and were first discovered in animals used the 2022 versions of the Swiss-Prot group’s ViralZone project (95) and the International Committee on Taxonomy of Viruses lists (96), as well as a 2018 paper on Classification of Human Viruses (54). Analyses of disease concerns in humans referenced WHO PHEIC declarations and Carlson et al.’s (47) Disease Outbreak News database, subset in 2023 for reports of only viral diseases, and then analyzed for the geographic and virological distribution of emerging disease threats. Noting surveillance biases, we report the frequency of DON reports as a *proxy* for both the occurrence and magnitude of a disease threat to reflect national and international public health institutions’ concerns regarding disease preparedness and response. The countermeasure analysis subset viral threats from four international and national priority pathogen lists created following the 2013 Ebola epidemic (WHO, NIAID [category A], UKVN, and CEPI). Across these data sets, except for DON reports, we reviewed the literature to ascertain the circumstances in which each virus was first isolated and the extent of subsequent outbreaks. We then used the framework described by Graham and Sullivan (97) to evaluate vaccine development across virus families. We conducted a literature review to determine, per family, when a vaccine was first approved for use in humans, and when and how the first virus currently associated with each family was isolated. For our filovirus case study, we based our literature search on the United States Centers for Disease Control and Prevention’s timeline of EVD and MVD outbreaks over time, with additional reports from WHO and regional health organizations. We reviewed the literature for studies that found samples positive for an *Orthoebolavirus* or *Orthomarburgvirus* by PCR or antigen test to characterize viral discovery in animals over time. In ascertaining whether an outbreak was caused by a novel spillover event, we assessed literature based on a schematic described in the Supplemental Methods. Finally, to determine the progression of various countermeasures through pre-clinical and clinical development, we searched PubMed for reviews of EVD and MVD countermeasure development using the search terms “ebola,” “marburg,” and “filovirus” with “countermeasures,” “vaccine,” “antibody,” and “antiviral” alongside search terms “ebola,” “marburg,” and “filovirus” on ClinicalTrials.gov. See Supplemental Methods for further detail.

## SUPPLEMENTAL MATERIAL

Data S1 mbio.02033-25-s0001.xlsx Source data for Fig. 3.

Data S2 mbio.02033-25-s0002.xlsx Source data for Fig. 3.

Data S3 mbio.02033-25-s0003.xlsx Source data for Fig. 3.

Data S4 mbio.02033-25-s0004.xlsx Source data for Fig. 4.

Data S5 mbio.02033-25-s0005.xlsx Source data for Fig. 4.

Data S6 mbio.02033-25-s0006.xlsx Source data for Fig. 4.

Supplemental material mbio.02033-25-s0007.docx Supplemental text and tables.

ASM does not own the copyrights to Supplemental Material that may be linked to, or accessed through, an article. The authors have granted ASM a non-exclusive, world-wide license to publish the Supplemental Material files. Please contact the corresponding author directly for reuse.
