# Supplementary material for: What is the relationship between viral prospecting in animals and medical countermeasure development?
Source: mBio. 2025 Aug 25;16(10):e02033-25. doi: 10.1128/mbio.02033-25 (PMC12506108; doi:10.1128/mbio.02033-25)
Supplement: Supplemental material — Supplemental text and tables. [file mbio.02033-25-s0007.docx]

**Supplementary Information**

**What is the relationship between viral prospecting in animals and medical countermeasure development?**

Aishani V. Aatresh and Marc Lipsitch

Table of Contents

2: Supplemental Methods

5: Table S1 (for Table 1)

7: Table S2 (for Figure 1)

9: Table S3 (for Table 2)

10: Table S4 (for Figure 2)

14: Table S5 (for Table 3)

20: Figures 3 and 4 information about supporting data and references

**Supplemental Methods**

To broadly assess relationships between discovery in animals, disease outbreaks in humans, and preparedness through medical countermeasures across viral taxa, we began by referencing 2022 versions of the ViralZone project^1^, managed by the virus program of the [Swiss-Prot group](https://www.sib.swiss/alan-bridge-group) of the [SIB Swiss Institute of Bioinformatics](https://www.sib.swiss/), and International Committee on Taxonomy of Viruses (ICTV) lists^2^, as well as a 2018 paper on Classification of Human Viruses^3^. We first assessed the ViralZone list for viruses that were isolated in animals prior to any subsequent clusters of cases in humans, excluding viruses for which only singleton human cases have been reported to focus on pathogens of public health concern for potential outbreaks. We also used these lists to determine the first recorded outbreak of any virus from each family with at least one virus known to infect humans, either by conducting that search or systematically evaluating each virus known to infect humans in each family.

We approached the question of viral discovery once more by analyzing documented outbreaks in humans. We subset Carlson et al.’s^4^ Disease Outbreak News database in 2023 to only include reports of viral diseases and then analyzed the geographic and virological distribution of emerging disease threats. We report frequency of DON, noting that extended outbreaks are represented with more frequent and recurring weekly reports. As a result, our visualization of DON offers a *proxy* for both the occurrence of a disease threat and the magnitude of it, which we chose over number of cases or disability-adjusted life years to reflect national and international public health institutions’ concerns regarding disease preparedness and response rather than epidemiological or economic statistics alone. DON nevertheless only includes clusters of diseases that are “large enough” in duration and scope, where a report of the cause and event fundamentally requires diagnostic capacity. (The greater number of Lassa fever reports in Europe, mainly imported cases, compared to Africa offers one such example.) Carlson et al. also note the limitations of this dataset due to considerations that include underreporting for some endemic diseases, unidentified novel threats, resource constraints, and changing standards for reports, it nonetheless offers an important and relatively localized snapshot of disease concerns in countries around the world. This limitation should be recognized when reviewing WHO and DON databases to assess for ‘new’ pathogens. We note the gaps in this dataset but are not focused on reconstructing an exact record of disease outbreaks over time and instead include this analysis as one way to account for more local disease reports and concerns. We then referenced a WHO list of Public Health Emergency of International Concern (PHEIC) declarations and performed a similar analysis of disease origins. PHEIC declarations also involve their own limitations; the system only has been in place since 2005, decisions to *not* declare a PHEIC for some outbreaks remain highly contentious, and zoonotic viruses like avian influenza remain highly consequential but have not been addressed through a PHEIC. Therefore, DON and PHEIC reports offer two partial but complementary ways to understand concerns about disease threats at different scales, with questions about viral discovery and countermeasure development in mind.

Our analysis of priority pathogens drew from the 4 lists created by international and national bodies following the 2013-2016 Ebola epidemic, namely: WHO, NIAID (Category A), UKVN, and CEPI. We compiled priority pathogens across these lists to focus on viral threats and then conducted similar reviews to ascertain the circumstances in which each virus was first isolated. The figure excludes smallpox, which only the NIAID lists prioritizes and on biosecurity grounds, because WHO declared the virus eradicated in 1980 and the first of several vaccines against the virus was developed in 1796. Furthemore, NIAID prioritizes viruses by family, listing several only for arenaviruses (Junin, Machupo, Guanarito, Chapare, Lassa, Lujo) as opposed to 1-3 for other families. The other lists only prioritize Lassa among potential arenaviruses. In this context and for economy, the figure only displays Lassa fever. Finally, the NIAID Category A list is current as of December 2023. In 2024, the NIAID prioritization list changed to omit biosafety-based categorizations and to include a much more expansive list of pathogens, which lends further support to the conclusion that knowledge of pathogenic threats is not a limiting “reagent” in MCM development (i.e., their very prioritization reflects a vast pipeline for potential work; see condition “v” in the Introduction).

We then used the virus families framework as described by Graham and Sullivan^5^, among others, to evaluate the vaccine development landscape across virus families. We conducted a literature review to determine the year in which a vaccine was first approved for use in humans, primarily by a regulatory body — with the exception of rabies and smallpox, for which widespread vaccination preceded any regulatory approval and initial vaccines did not involve the isolation of either virus as such^6,7^. We coupled our evaluation of vaccine development with our analyses of when and how the first virus associated with each family was isolated, irrespective of the specific virus’ ability to infect humans. We focused on vaccines as opposed to other medical countermeasures because of our focus on public health approaches to preparedness over treatment in clinical settings, although we acknowledge the importance of therapeutic and diagnostic development as other relevant aspects for future consideration.

For our filoviruses case study, we based our literature search on information from the United States Centers for Disease Control and Prevention’s (CDC) timeline of EVD and MVD outbreaks over time. We used this timeline to then expand data collection to World Health Organizational and sub-regional organizations’ outbreak disease reports for additional epidemiological information. These sources were used to find key publications through PubMed and article citations to answer specific questions surrounding evidence for novel spillover and general outbreak origins, the context within which an outbreak began, and the demographics within which the virus spread. Case counts for each outbreak are based on laboratory-confirmed and suspected cases, where possible. In ascertaining whether an outbreak was caused by a novel spillover event, we used the following schematic: 1) *True* if both genomic and epidemiological evidence supported a novel spillover event, given availability of genomic data; 2) *Probable* if relatively conclusive genomic or epidemiological evidence but lacking evidence of the other form (provided both methods were available) or some other acknowledgement of uncertainty; 3) *Disputed/Possible* with competing explanations and unresolved debates within the literature; 4) *Unknown* if there was no evidence to whether a novel spillover event or existing circulating infection could be identified as the source of the outbreak or if literature generally states that the origins of a particular outbreak are unknown; 5) *False* if both genomic and epidemiological evidence identified existing and circulating infections in humans as the outbreak source. We note that the success of investigations into spillover events can be limited by the time delay between an undetected first spillover event, detection of cases in people, and the start of an investigation. We conducted literature review for studies that found samples positive for an *Orthoebolavirus* or *Orthomarburgvirus* by polymerase chain reaction or antigen test to characterize animal host discovery over time, corroborated by GenBank searches for sequencing data. We did not include studies that reported seropositivity for either filovirus because this analysis focuses on surveillance for viruses to further understandings of potential pathogens rather than presence or absence of known viruses that elucidate, for instance, traces of infection in animal hosts. These criteria exclude several studies in which animal hosts are seropositive for a filovirus but none are positive by a molecular test for the presence of virus. For similar reasons, we did not include the many studies that describe efforts that failed to detect any sample that was positive for an *Orthoebolavirus* or *Orthomarburgvirus*. Finally, we searched PubMed for reviews of EVD and MVD countermeasure development using the search terms “ebola,” “marburg,” and “filovirus” with “countermeasures,” “vaccine,” “antibody,” and “antiviral.” These reviews guided the construction of a set of relevant MCMs of interest, which then informed a further literature search to ascertain the timeline of clinical development (e.g., not basic research) for these countermeasures. The lack of any clinical trial beyond Phase I for MVD motivated further characterization of preclinical research against Marburg viruses. In parallel, the search terms “ebola,” “marburg,” and “filovirus” were used on ClinicalTrials.gov to determine the progression of various countermeasures through clinical trials. Our dataset primarily includes the first instance of a clinical trial in a particular phase for a particular vaccine or drug candidate and is not comprehensive as to include all trials for comparing different dosage regimes or studying efficacy in different populations, for example.

1. Swiss-Prot. Human viruses and associated pathologies. *ViralZone | SIB Swiss Institute of Bioinformatics* https://viralzone.expasy.org/678 (2024).

2. Master Species Lists | ICTV. https://ictv.global/msl.

3. Siegel, R. D. Classification of Human Viruses. *Principles and Practice of Pediatric Infectious Diseases* 1044-1048.e1 (2018) doi:10.1016/B978-0-323-40181-4.00201-2.

4. Carlson, C. J. *et al.* The World Health Organization’s Disease Outbreak News: A retrospective database. *PLOS Global Public Health* **3**, e0001083 (2023).

5. Graham, B. S. & Sullivan, N. J. Emerging viral diseases from a vaccinology perspective: preparing for the next pandemic. *Nat Immunol* **19**, 20–28 (2018).

6. Rupprecht, C. E., Glickman, L. T., Spencer, P. A. & Wiktor, T. J. EPIDEMIOLOGY OF RABIES VIRUS VARIANTS: DIFFERENTIATION USD MONOCLONAL ANTIBODIES AND DISCRIMINANT ANALYSIS. *American Journal of Epidemiology* **126**, 298–309 (1987).

7. McCollum, A. M. *et al.* Poxvirus Viability and Signatures in Historical Relics. *Emerg. Infect. Dis.* **20**, 177–184 (2014).

**Table S1: Viruses first discovered in animals before causing an outbreak in humans**

*Corresponds with Table 1*

| **Virus** | **References** |
| --- | --- |
| Barmah Forest | 1 |
| Bunyamwera | 2, 3 |
| Eastern equine encephalitis | 4 |
| Monkeypox | 5 |
| Ngari | 2, 6 |
| Puumala | 7 |
| Rift Valley Fever | 8 |
| Semliki Forest | 9, 10, 11 |
| Sindbis | 12, 13 |
| Venezuelan equine encephalitis | 14 |
| Zika | 15 |

1. Lindsay, M. D. A., Johansen, C. A., Broom, A. K., Smith, D. W. & MacKenzie, J. S. Emergence of Barmah Forest Virus in Western Australia - Volume 1, Number 1—January 1995 - Emerging Infectious Diseases journal - CDC. doi:10.3201/eid0101.950104.
2. Smithburn, K. C., Paterson, H. E., Kokernot, R. H. & De Meillon, B. Isolation of Bunyamwera Virus from a Naturally Infected Human Being and Further Isolations from Aedes (Banksinella) Circumluteolus Theo. 1. *The American Journal of Tropical Medicine and Hygiene* **7**, 579–584 (1958).
3. Dutuze, M. F., Nzayirambaho, M., Mores, C. N. & Christofferson, R. C. A Review of Bunyamwera, Batai, and Ngari Viruses: Understudied Orthobunyaviruses With Potential One Health Implications. *Front. Vet. Sci.* **5**, 69 (2018).
4. Corrin, T., Ackford, R., Mascarenhas, M., Greig, J. & Waddell, L. A. Eastern Equine Encephalitis Virus: A Scoping Review of the Global Evidence. *Vector Borne Zoonotic Dis.* **21**, 305–320 (2021).
5. World Health Organization. Mpox (monkeypox). https://www.who.int/news-room/fact-sheets/detail/monkeypox (2023).
6. Zeller, H. G. et al. [Ngari virus (Bunyaviridae: Bunyavirus). First isolation from humans in Senegal, new mosquito vectors, its epidemiology]. *Bull Soc Pathol Exot* **89**, 12–16 (1996).
7. Castel, G. et al. Phylogeography of Puumala orthohantavirus in Europe. *Viruses* **11**, 679 (2019).
8. European Centre for Disease Prevention and Control. Facts about Rift Valley fever. https://www.ecdc.europa.eu/en/rift-valley-fever/facts (2010).
9. Grimaud, G. et al. An Outbreak of Human Semliki Forest Virus Infections in Central African Republic. *The American Journal of Tropical Medicine and Hygiene* **42**, 386–393 (1990).
10. Smithburn, K. C. & Haddow, A. J. Semliki Forest Virus. *The Journal of Immunology* **49**, 141–157 (1944).
11. Smithburn, K. C., Haddow, A. J. & Mahaffy, A. F. A Neurotropic Virus Isolated from Aedes Mosquitoes Caught in the Semliki Forest. *The American Journal of Tropical Medicine and Hygiene* s1-26, 189–208 (1946).
12. Meno, K., Yah, C., Mendes, A. & Venter, M. Incidence of Sindbis Virus in Hospitalized Patients With Acute Fevers of Unknown Cause in South Africa, 2019–2020. *Front. Microbiol.* **12**, 798810 (2022).
13. Atkins, G. J., Sheahan, B. J. & Liljeström, P. The molecular pathogenesis of Semliki Forest virus: a model virus made useful? *Journal of General Virology* **80**, 2287–2297 (1999).
14. Aguilar, P. V. et al. Endemic Venezuelan equine encephalitis in the Americas: hidden under the dengue umbrella. *Future Virol.* **6**, 721–740 (2011).
15. World Health Organization. The history of zika virus. https://www.who.int/news-room/feature-stories/detail/the-history-of-zika-virus (2016).

Note Bunyamwera virus is listed along with Cache Valley virus (CVV) in the Swiss-Prot classification scheme, while other sources list CVV as a novel species which was discovered in non-human animals. See: <https://www.niaid.nih.gov/research/niaid-biodefense-pathogens>; <https://academic.oup.com/jme/article/60/6/1230/7321677>

**Table S2: Viral disease events in DON database**

*Corresponds with Figure 1: Geographic and viral distribution of WHO DON reports (1996-2019)*

| **Disease** | **PathogenType** |
| --- | --- |
| Anthrax |  |
| Botulism |  |
| Buffalopox | Virus |
| Chikungunya | Virus |
| Cholera |  |
| Coccidioidomycosis | |
| Crimean-Congo haemorrhagic fever | Virus |
| Dengue fever | Virus |
| Diptheria |  |
| Dysentery |  |
| E. coli |  |
| Ebola virus | Virus |
| Elizabethkingia anophelis | |
| Enterovirus | Virus |
| Gonorrhea |  |
| Guillain-Barre syndrome | |
| Hand, foot, and mouth disease | Virus |
| Hantavirus | Virus |
| Hemolytic uremic syndrome | |
| Hepatitis A | Virus |
| Hepatitis E | Virus |
| Human coronavirus OC43 | Virus |
| Human immunodeficiency virus | Virus |
| Influenza A | Virus |
| Japanese encephalitis | Virus |
| Lassa fever | Virus |
| Legionellosis | |
| Leishmaniasis | |
| Leptospirosis | |
| Listeriosis |  |
| Lujo mammarenavirus | Virus |
| Malaria |  |
| Marburg fever | Virus |
| Measles | Virus |
| Meningococcal disease | |
| MERS-CoV | Virus |
| Monkeypox | Virus |
| Nipah virus | Virus |
| O'nyong-nyong fever | Virus |
| Oropouche fever | Virus |
| Pertussis |  |
| Plague |  |
| Polio | Virus |
| Pseudomonas aeruginosa | |
| Rabies | Virus |
| Rift Valley fever | Virus |
| Salmonella enterica | |
| SARS-CoV | Virus |
| Smallpox | Virus |
| St. Louis encephalitis | Virus |
| Staphylococcus | |
| Streptococcus suis | |
| Syndromic: cardiovascular | |
| Syndromic: diarrhoeal | |
| Syndromic: gastrointestinal | |
| Syndromic: haemorrhagic | |
| Syndromic: neurological | |
| Syndromic: respiratory | |
| Tick-borne relapsing fever | |
| Toxicity: bromide poisoning | |
| Toxicity: lead poisoning | |
| Toxicity: miscellaneous | |
| Transmissible spongiform encephalopathy | |
| Tuberculosis | |
| Tularemia |  |
| Typhoid |  |
| Typhus |  |
| Unspecified |  |
| Venezuelan equine encephalitis | Virus |
| West Nile virus | Virus |
| Yellow fever | Virus |
| Zika virus disease | Virus |

**Table S3: WHO PHEIC declarations** (*Corresponds with Table 2)*

| **Year** | **Viral Disease** | **Novel Pathogen?** | **Notes** | **References** |
| --- | --- | --- | --- | --- |
| 2009 | H1N1 | TRUE |  | 1, 2 |
| 2014 | Ebola |  |  | 1, 3 |
| 2014 | Polio |  | PHEIC is ongoing | 1, 4 |
| 2016 | Zika |  |  | 1, 5 |
| 2018 | Ebola |  |  | 1, 6 |
| 2020 | COVID-19 | TRUE |  | 1, 7 |
| 2022 | mpox |  |  | 8, 9 |

1. Wilder-Smith, A. & Osman, S. Public health emergencies of international concern: a historic overview. *J Travel Med* **27**, taaa227 (2020).
2. H1N1 IHR Emergency Committee. *World Health Organization* https://www.who.int/groups/h1n1-ihr-emergency-committee (2010).
3. Ebola outbreak 2014-2016 - West Africa. *World Health Organization* https://www.who.int/emergencies/situations/ebola-outbreak-2014-2016-West-Africa (2024).
4. Public Health Emergency status. *Global Polio Eradication Initiative* https://polioeradication.org/polio-today/polio-now/public-health-emergency-status/ (2024).
5. Yakob, L. Zika Virus after the Public Health Emergency of International Concern Period, Brazil. *Emerg. Infect. Dis.* **28**, 837–840 (2022).
6. Ebola outbreak in the Democratic Republic of the Congo declared a Public Health Emergency of International Concern. *World Health Organization* https://www.who.int/news/item/17-07-2019-ebola-outbreak-in-the-democratic-republic-of-the-congo-declared-a-public-health-emergency-of-international-concern (2019).
7. COVID-19 Public Health Emergency of International Concern (PHEIC) Global research and innovation forum. *World Health Organization* https://www.who.int/publications/m/item/covid-19-public-health-emergency-of-international-concern-(pheic)-global-research-and-innovation-forum (2020).
8. Mpox (monkeypox) outbreak 2022. *World Health Organization* https://www.who.int/emergencies/situations/monkeypox-oubreak-2022 (2024).
9. Major Epidemics of the Modern Era. *Council on Foreign Relations* https://www.cfr.org/timeline/major-epidemics-modern-era (2023).

| **Viral Pathogen/**  **Family** | **List** | **Year Isolated** | **How Discovered?** | **Zoonotic Transmission** | **Detection Detail** | **Notes** | **References** |
| --- | --- | --- | --- | --- | --- | --- | --- |
| MERS | CEPI, WHO, UK | 2012 | patient | TRUE | first human outbreak |  | 1, 2 |
| Lassa Fever | CEPI, UK, NIAID | 1969 | animal + patient | TRUE | first human outbreak | Some outbreaks reported in the 1950s. | 3 |
| Nipah | CEPI, UK | 1999 | patient | TRUE | first human outbreak |  | 4 |
| Rift Valley Fever | CEPI, WHO, UK | 1931 | animal | TRUE | pre human outbreak | Until 1975, RVF was regarded as an African, animal disease. Human cases were rare and with mild clinical manifestations. | 5 |
| Chikungunya | CEPI, UK | 1953 | patient | *vector-borne | during human outbreak |  | 6 |
| Ebola | CEPI, WHO, UK, NIAID | 1976 | patient | TRUE | first human outbreak |  | 7 |
| Marburg | WHO, UK, NIAID | 1967 | patient | TRUE | first human outbreak | in Europe, non endemic | 8 |
| COVID-19 | WHO | 2020 | patient | TRUE | first human outbreak |  | 9 |
| CCHF | WHO, UK | 1956 | patient | *vector-borne | during human outbreak | Disease Crimean Peninsula. Virus isolated Later Congo Basin. | 10, 11 |
| SARS | WHO | 2003 | patient | TRUE | first human outbreak |  | 12 |
| Henipaviruses | WHO | 1994 | animal + patient | TRUE | during human outbreak | Hendra virus | 13 |
| Zika | WHO, UK | 1947 | animal | *vector-borne | pre human outbreak | 1947 mosquitoes, 1952 humans. Found through yellow fever surveillance. First proof of human disease 1964. | 14 |
| Dengue | UK, NIAID | 1943 | patient | *vector-borne | during human outbreak | NIAID focuses on flaviviruses at the family level. | 15 |
| Hantaviruses | UK, NIAID | 1978 | animal | TRUE | post human outbreak | NIAID focuses on bunyaviruses at the family level, of which hantaviruses are one example. | 16 |
| Smallpox | NIAID | 1796 | patient | FALSE | during human outbreak | vaccine in 1796, Edward Jenner. Excluded from figure because i) existing vaccine with regulatory approval ii) disease eradicated iii) biosecurity concern specific to NIAID | 17 |

1. Middle East respiratory syndrome coronavirus (MERS-CoV). *World Health Organization* https://www.who.int/news-room/fact-sheets/detail/middle-east-respiratory-syndrome-coronavirus-(mers-cov) (2022).
2. Zumla, A., Hui, D. S. & Perlman, S. Middle East respiratory syndrome. *The Lancet* **386**, 995–1007 (2015).
3. Monath, T. P., Newhouse, V. F., Kemp, G. E., Setzer, H. W. & Cacciapuoti, A. Lassa Virus Isolation from *Mastomys natalensis* Rodents during an Epidemic in Sierra Leone. *Science* **185**, 263–265 (1974).
4. Ang, B. S. P., Lim, T. C. C. & Wang, L. Nipah Virus Infection. *J Clin Microbiol* **56**, e01875-17 (2018).
5. Facts about Rift Valley fever. *European Centre for Disease Prevention and Control* https://www.ecdc.europa.eu/en/rift-valley-fever/facts (2010).
6. Ross, R. W. The Newala epidemic: III. The virus: isolation, pathogenic properties and relationship to the epidemic. *J. Hyg.* **54**, 177–191 (1956).
7. Breman, J. G. *et al.* Discovery and Description of Ebola Zaire Virus in 1976 and Relevance to the West African Epidemic During 2013–2016. *J Infect Dis* **214**, S93–S101 (2016).
8. Marburg virus disease. *World Health Organization* https://www.who.int/news-room/fact-sheets/detail/marburg-virus-disease (2021).
9. Archived: WHO Timeline - COVID-19. *World Health Organization* https://www.who.int/news/item/27-04-2020-who-timeline---covid-19 (2020).
10. Factsheet about Crimean-Congo haemorrhagic fever. *European Centre for Disease Prevention and Control* https://www.ecdc.europa.eu/en/crimean-congo-haemorrhagic-fever/facts/factsheet (2017).
11. Mourya, D. T. *et al.* Detection, Isolation and Confirmation of Crimean-Congo Hemorrhagic Fever Virus in Human, Ticks and Animals in Ahmadabad, India, 2010–2011. *PLoS Negl Trop Dis* **6**, e1653 (2012).
12. Shi, Z. & Wang, L.-F. Evolution of SARS Coronavirus and the Relevance of Modern Molecular Epidemiology. *Genetics and Evolution of Infectious Diseases* 601–619 (2017) doi:10.1016/B978-0-12-799942-5.00026-3.
13. Marsh, G. A. *et al.* Genome Sequence Conservation of Hendra Virus Isolates during Spillover to Horses, Australia. *Emerg Infect Dis* **16**, 1767–1769 (2010).
14. Factsheet about Zika virus disease. *European Centre for Disease Prevention and Control* https://www.ecdc.europa.eu/en/zika-virus-infection/facts/factsheet (2017).
15. San Martín, J. L. *et al.* The History of Dengue Outbreaks in the Americas. *The American Journal of Tropical Medicine and Hygiene* **87**, 584–593 (2012).
16. Mir, M. Hantaviruses. *Clin Lab Med* **30**, 67–91 (2010).
17. Sánchez-Sampedro, L. *et al.* The Evolution of Poxvirus Vaccines. *Viruses* **7**, 1726–1803 (2015).

***Table S5: Vaccine development and viral discovery across virus families***

Corresponds with Table 3

| **Family** | **References** |
| --- | --- |
| Adenoviridae | 1, 2, 3 |
| Anelloviridae | 4, 5 |
| Arenaviridae | 6, 7, 8, 9 |
| Astroviridae | 10, 11, 12 |
| Bornaviridae | 13, 14, 15, 16 |
| Bunyaviridae | 17, 18 |
| Caliciviridae | 19, 20, 21 |
| Coronaviridae | 22 |
| Filoviridae | 8, 23 |
| Flaviviridae | 24, 25, 26 |
| Hepadnaviridae | 27, 28 |
| Hepeviridae | 29, 30, 71 |
| Herpesviridae | 31 |
| Orthomyxoviridae | 32 |
| Papillomaviridae | 33, 34, 35, 36, 37 |
| Paramyxoviridae | 38, 39, 40 |
| Parvoviridae | 41, 42, 43 |
| Picobirnaviridae | 44 |
| Picornaviridae | 45, 46, 47 |
| Pneumoviridae | 48, 49, 50 |
| Polyomaviridae | 51, 52, 53 |
| Poxviridae | 54, 55, 56 |
| Reoviridae | 57, 58, 59, 60 |
| Retroviridae | 61, 62 |
| Rhabdoviridae | 63, 64, 65 |
| Togaviridae | 66, 67, 68, 69, 70 |

1. Centers for Disease Control and Prevention. Adenovirus Vaccine Information Statement. *Vaccine Information Statements* https://www.cdc.gov/vaccines/hcp/vis/vis-statements/adenovirus.html (2020).
2. Soares, J. M. USAMMDA Seeks Refresh of Adenovirus Vaccine. *www.army.mil* https://www.army.mil/article/183287/usammda_seeks_refresh_of_adenovirus_vaccine (2017).
3. Robinson, C. M., Seto, D., Jones, M. S., Dyer, D. W. & Chodosh, J. Molecular evolution of human species D adenoviruses. *Infect Genet Evol* **11**, 1208–1217 (2011).
4. Harnessing the Untapped Potential of Anelloviruses. *Flagship Pioneering* https://www.flagshippioneering.com/stories/harnessing-the-untapped-potential-of-anelloviruses (2022).
5. Spezia, P. G. *et al.* TTV and other anelloviruses: The astonishingly wide spread of a viral infection. *Asp Mol Med* **1**, None (2023).
6. Carnec, X. *et al.* A Vaccine Platform against Arenaviruses Based on a Recombinant Hyperattenuated Mopeia Virus Expressing Heterologous Glycoproteins. *J Virol*  **92**, e02230-17 (2018).
7. Gowen, B. B. *et al.* Second-Generation Live-Attenuated Candid#1 Vaccine Virus Resists Reversion and Protects against Lethal Junín Virus Infection in Guinea Pigs. *J Virol* **95**, e0039721 (2021).
8. CDC. About Viral Hemorrhagic Fevers. *Viral Hemorrhagic Fevers (VHFs)* https://www.cdc.gov/viral-hemorrhagic-fevers/about/index.html (2024).
9. Johnson, D. M. *et al.* Bivalent Junin & Machupo experimental vaccine based on alphavirus RNA replicon vector. *Vaccine* **38**, 2949–2959 (2020).
10. Bosch, A., Pintó, R. M. & Guix, S. Human Astroviruses. *Clin Microbiol Rev* **27**, 1048–1074 (2014).
11. Human Astrovirus Vaccine. *Creative Biolabs* https://www.creative-biolabs.com/vaccine/human-astrovirus-vaccine.htm.
12. Bidokhti, M. R. M. *et al.* Immunogenicity and Efficacy Evaluation of Subunit Astrovirus Vaccines. *Vaccines (Basel)***7**, 79 (2019).
13. Dürrwald, R. *et al.* Vaccination against Borna Disease: Overview, Vaccine Virus Characterization and Investigation of Live and Inactivated Vaccines. *Viruses* **14**, 2706 (2022).
14. Honda, T. Relaunching human bornavirus research from encephalitis cases with unclear cause. *The Lancet Infectious Diseases* **20**, 389–391 (2020).
15. Bauswein, M. *et al.* Human Infections with Borna Disease Virus 1 (BoDV-1) Primarily Lead to Severe Encephalitis: Further Evidence from the Seroepidemiological BoSOT Study in an Endemic Region in Southern Germany. *Viruses* **15**, 188 (2023).
16. Tizard, I., Ball, J., Stoica, G. & Payne, S. The pathogenesis of bornaviral diseases in mammals. *Animal Health Research Reviews* **17**, 92–109 (2016).
17. Ikegami, T. & Makino, S. Rift Valley fever vaccines. *Vaccine* **27S4**, D69–D72 (2009).
18. Wichgers Schreur, P. J., Bird, B. H., Ikegami, T., Bermúdez-Méndez, E. & Kortekaas, J. Perspectives of Next-Generation Live-Attenuated Rift Valley Fever Vaccines for Animal and Human Use. *Vaccines (Basel)* **11**, 707 (2023).
19. Feline Calicivirus. *Cornell University College of Veterinary Medicine* https://www.vet.cornell.edu/departments-centers-and-institutes/baker-institute/research-baker-institute/feline-calicivirus.
20. Tan, M. Norovirus Vaccines: Current Clinical Development and Challenges. *Pathogens* **10**, 1641 (2021).
21. Caliciviridae. *Encyclopedia of Virology (Third Edition)* https://www.sciencedirect.com/topics/medicine-and-dentistry/caliciviridae (2008).
22. Krammer, F. The role of vaccines in the COVID-19 pandemic: what have we learned? *Semin Immunopathol* **45**, 451–468 (2024).
23. First FDA-approved vaccine for the prevention of Ebola virus disease, marking a critical milestone in public health preparedness and response. *U.S. Food & Drug Administration* https://www.fda.gov/news-events/press-announcements/first-fda-approved-vaccine-prevention-ebola-virus-disease-marking-critical-milestone-public-health (2019).
24. Tien, S.-M. *et al.* Therapeutic efficacy of humanized monoclonal antibodies targeting dengue virus nonstructural protein 1 in the mouse model. *PLOS Pathogens* **18**, e1010469 (2022).
25. Norrby, E. Yellow fever and Max Theiler: the only Nobel Prize for a virus vaccine. *J Exp Med* **204**, 2779–2784 (2007).
26. Gianchecchi, E., Cianchi, V., Torelli, A. & Montomoli, E. Yellow Fever: Origin, Epidemiology, Preventive Strategies and Future Prospects. *Vaccines (Basel)* **10**, 372 (2022).
27. Hepadnaviridae. *ScienceDirect | International Journal of Infectious Diseases* https://www.sciencedirect.com/topics/medicine-and-dentistry/hepadnaviridae (2008).
28. CDC. Chapter 10: Hepatitis B. *Epidemiology and Prevention of Vaccine-Preventable Diseases* https://www.cdc.gov/pinkbook/hcp/table-of-contents/chapter-10-hepatitis-b.html (2024).
29. Kelly, A. G., Netzler, N. E. & White, P. A. Ancient recombination events and the origins of hepatitis E virus. *BMC Evolutionary Biology* **16**, 210 (2016).
30. Sridhar, S. *et al.* A Systematic Approach to Novel Virus Discovery in Emerging Infectious Disease Outbreaks. *J Mol Diagn* **17**, 230–241 (2015).
31. World Health Organization. Herpes Simplex Virus. *Immunization, Vaccines and Biologicals* https://www.who.int/teams/immunization-vaccines-and-biologicals/diseases/herpes-simplex-virus (2022).
32. History of influenza vaccination. *World Health Organization* https://www.who.int/news-room/spotlight/history-of-vaccination/history-of-influenza-vaccination.
33. CDC. HPV Vaccination. *Human Papillomavirus (HPV)* https://www.cdc.gov/hpv/vaccines/index.html (2024).
34. KFF. The HPV Vaccine: Access and Use in the U.S. *Women’s Health Policy* https://www.kff.org/womens-health-policy/fact-sheet/the-hpv-vaccine-access-and-use-in-the-u-s/ (2021).
35. Dürst, M., Gissmann, L., Ikenberg, H. & zur Hausen, H. A papillomavirus DNA from a cervical carcinoma and its prevalence in cancer biopsy samples from different geographic regions. *Proc Natl Acad Sci U S A* **80**, 3812–3815 (1983).
36. Watts, G. Harald zur Hausen. *The Lancet* **402**, 20 (2023).
37. The Nobel Prize in Physiology or Medicine 2008. *The Nobel Prize* https://www.nobelprize.org/prizes/medicine/2008/advanced-information/.
38. Hendriks, J. & Blume, S. Measles Vaccination Before the Measles-Mumps-Rubella Vaccine. *Am J Public Health* **103**, 1393–1401 (2013).
39. Almansour, I. Mumps Vaccines: Current Challenges and Future Prospects. *Front Microbiol* **11**, 1999 (2020).
40. Johnson, C. D. & Goodpasture, E. W. AN INVESTIGATION OF THE ETIOLOGY OF MUMPS. *J Exp Med* **59**, 1–19 (1934).
41. Bernstein, D. I. *et al.* Safety and Immunogenicity of a Candidate Parvovirus B19 Vaccine. *Vaccine* **29**, 7357–7363 (2011).
42. Pénzes, J. J. *et al.* Reorganizing the family Parvoviridae: a revised taxonomy independent of the canonical approach based on host association. *Arch Virol* **165**, 2133–2146 (2020).
43. Union of International Associations. Parvoviruses. *THE ENCYCLOPEDIA OF WORLD PROBLEMS & HUMAN POTENTIAL* http://encyclopedia.uia.org/en/problem/parvoviruses.
44. Wang, D. The enigma of picobirnaviruses: viruses of animals, fungi, or bacteria? *Curr Opin Virol* **54**, 101232 (2022).
45. Polio Vaccination. *CDC* https://www.cdc.gov/vaccines/vpd/polio/index.html (2022).
46. History of polio vaccination. *World Health Organization* https://www.who.int/news-room/spotlight/history-of-vaccination/history-of-polio-vaccination.
47. Berhanu, A. PICORNAVIRIDAE. https://web.stanford.edu/group/virus/picorna/2005/PICORNAVIRIDAE.htm.
48. CDC. RSV Vaccine Information Statement. https://www.cdc.gov/vaccines/hcp/vis/vis-statements/rsv.html (2023).
49. FDA Approves First Respiratory Syncytial Virus (RSV) Vaccine. https://www.fda.gov/news-events/press-announcements/fda-approves-first-respiratory-syncytial-virus-rsv-vaccine (2023).
50. Collins, P. L. & Graham, B. S. Viral and Host Factors in Human Respiratory Syncytial Virus Pathogenesis. *J Virol***82**, 2040–2055 (2008).
51. Peretti, A. *et al.* A multivalent polyomavirus vaccine elicits durable neutralizing antibody responses in macaques. *Vaccine* **41**, 1735–1742 (2023).
52. Neu, U., Stehle, T. & Atwood, W. J. The Polyomaviridae: Contributions of virus structure to our understanding of virus receptors and infectious entry. *Virology* **384**, 389–399 (2009).
53. IARC Working Group on the Evaluation of Carcinogenic Risks to Humans. INTRODUCTION TO POLYOMAVIRUSES. in *Malaria and Some Polyomaviruses (SV40, BK, JC, and Merkel Cell Viruses)* (International Agency for Research on Cancer, 2013).
54. Sánchez-Sampedro, L. *et al.* The Evolution of Poxvirus Vaccines. *Viruses* **7**, 1726–1803 (2015).
55. History of Smallpox | Smallpox. *CDC* https://www.cdc.gov/smallpox/history/history.html (2024).
56. Thèves, C., Biagini, P. & Crubézy, E. The rediscovery of smallpox. *Clinical Microbiology and Infection* **20**, 210–218 (2014).
57. Rotavirus Vaccination. *CDC* https://www.cdc.gov/vaccines/vpd/rotavirus/index.html (2023).
58. CDC. Chapter 19: Rotavirus. *Epidemiology and Prevention of Vaccine-Preventable Diseases*https://www.cdc.gov/pinkbook/hcp/table-of-contents/chapter-19-rotavirus.html (2024).
59. Kim, M. Naturally occurring reoviruses for human cancer therapy. *BMB Rep* **48**, 454–460 (2015).
60. Sabin, A. B. Reoviruses: A new group of respiratory and enteric viruses formerly classified as ECHO type 10 is described. *Science* **130**, 1387–1389 (1959).
61. Coffin, J. M., Hughes, S. H. & Varmus, H. E. A Brief Chronicle of Retrovirology. in *Retroviruses* (Cold Spring Harbor Laboratory Press, 1997).
62. Vahlne, A. A historical reflection on the discovery of human retroviruses. *Retrovirology* **6**, 40 (2009).
63. Hicks, D. J., Fooks, A. R. & Johnson, N. Developments in rabies vaccines. *Clin Exp Immunol* **169**, 199–204 (2012).
64. Fooks, A. R., Banyard, A. C. & Ertl, H. C. J. New human rabies vaccines in the pipeline. *Vaccine* **37**, A140–A145 (2019).
65. Rabies Vaccine Information Statement. *CDC* https://www.cdc.gov/vaccines/hcp/vis/vis-statements/rabies.html (2023).
66. About Rubella (German Measles) Vaccination. *CDC* https://www.cdc.gov/vaccines/vpd/rubella/index.html (2022).
67. Western Equine Encephalitis. *ScienceDirect* https://www.sciencedirect.com/topics/medicine-and-dentistry/western-equine-encephalitis.
68. CDC. Chapter 20: Rubella. *Epidemiology and Prevention of Vaccine-Preventable Diseases*https://www.cdc.gov/pinkbook/hcp/table-of-contents/chapter-20-rubella.html (2024).
69. History and Timeline. https://web.stanford.edu/group/virus/1999/jchow/histtime.html (1999).
70. Brinton, M. A. Replication of Flaviviruses. in *The Togaviridae and Flaviviridae* (eds. Schlesinger, S. & Schlesinger, M. J.) 327–374 (Springer New York, Boston, MA, 1986). doi:10.1007/978-1-4757-0785-4_11.
71. Huang, X. *et al.* Progress and Challenges to Hepatitis E Vaccine Development and Deployment. *Vaccines* **12**, 719 (2024).

.xlsx files indicate sources for data in the following figures:

***Figure 3: Marburg outbreaks, animal surveillance, and MCM development***

***Figure 4: Ebola outbreaks, animal surveillance, and MCM development***
